# Repetitive transcranial magnetic stimulation therapy for motor recovery in Parkinson's disease: A Meta‐analysis

**DOI:** 10.1002/brb3.1132

**Published:** 2018-09-28

**Authors:** Changxia Yang, Zhiwei Guo, Haitao Peng, Guoqiang Xing, Huaping Chen, Morgan A. McClure, Bin He, Lin He, Fei Du, Liangwen Xiong, Qiwen Mu

**Affiliations:** ^1^ Department of Radiology & Imaging Institute of Rehabilitation and Development of Brain Function The Second Clinical Medical College of North Sichuan Medical College Nanchong Central Hospital Nanchong China; ^2^ Chengdu 363 Hospital of Southwest Medical University Chengdu China; ^3^ Lotus Biotech.com LLC John Hopkins University‐MCC Rockville Maryland; ^4^ Department of Psychiatry Harvard Medical School Belmont Massachusetts; ^5^ Department of Genitourinary The University of Texas MD Anderson Cancer Center Houston Texas; ^6^ Peking University Third Hospital Beijing China

**Keywords:** meta‐analysis, motor function, Parkinson's disease, repetitive transcranial magnetic stimulation

## Abstract

**Introduction:**

Therapeutic effects of repetitive transcranial magnetic stimulation (rTMS) on motor recovery of Parkinson's disease (PD) have been reported; however, the protocols of these studies varied greatly. The aim of this meta‐analysis was to evaluate the optimal rTMS parameters for motor recovery of PD.

**Methods:**

Electronic databases were searched for studies investigating the therapeutic effects of rTMS on motor function in patients with PD. The section III of the Unified Parkinson's Disease Rating Scale (UPDRS) was extracted as the primary outcome, and the standardized mean difference (SMD) with 95% confidence interval (CI) was calculated.

**Results:**

Twenty‐three studies with a total of 646 participants were included. The pooled estimates of rTMS revealed significant short‐term (SMD, 0.37; *p* < 0.00001) and long‐term (SMD, 0.39; *p* = 0.005) effects on motor function improvement of PD. Subgroup analysis observed that high‐frequency rTMS (HF‐rTMS) was significant in improving motor function (SMD, 0.48; *p* < 0.00001), but low‐frequency rTMS (LF‐rTMS) was not. In particular, when HF‐rTMS targeted over the primary motor cortex (M1), in which the bilateral M1 revealed a larger effect size than unilateral M1. Compared to single‐session, multi‐session of HF‐rTMS over the M1 showed significant effect size. In addition, HF‐rTMS over the M1 with a total of 18,000–20,000 stimulation pulses yielded more significant effects (SMD, 0.97; *p* = 0.01) than other dosages.

**Conclusions:**

In conclusion, multi‐session of HF‐rTMS over the M1 (especially bilateral M1) with a total of 18,000–20,000 pulses appears to be the optimal parameters for motor improvement of PD.

## 1 INTRODUCTION

Parkinson's disease (PD) is the second most common neurodegenerative diseases worldwide (de Lau & Breteler, 2006) affecting about 6.2 million people globally in 2015 (Collaborators GMaCoD, 2016). Motor dysfunction of PD is mainly manifested as resting tremors, rigidity, bradykinesia, and postural instability (Jankovic, 2008). The loss of presynaptic nigrostriatal dopamine neurons and a progressive nigrostriatal dopamine deficiency in the cortico‐striato‐thalamocortical circuit is considered the primary mechanism of motor dysfunction of PD (Wichmann, DeLong, Guridi, & Obeso, 2011). Many therapeutic approaches have been developed for the treatment of PD.

Repetitive transcranial magnetic stimulation (rTMS), by its ability to modulate cortical excitability, is a safe and noninvasive therapy that has been widely used for the treatment of neurological and psychiatric disorders including stroke, Alzheimer's disease, depression, and PD (Chen et al., 2014; Hsu, Cheng, Liao, Lee, & Lin, 2012; Liao et al., 2015). High‐frequency rTMS (HF‐rTMS) (>1.0 Hz) can enhance the cortical excitability whereas low‐frequency (≤1.0 Hz) rTMS (LF‐rTMS) can decrease the cortical excitability (Maeda, Keenan, Tormos, Topka, & Pascual‐Leone, 2000; Peinemann et al., 2004). Theta burst stimulation (TBS) is another form of rTMS protocol with a high‐frequency and low‐intensity stimulation. Intermittent theta burst stimulation (iTBS) enhances cortical excitability, whereas continuous theta stimulation (cTBS) decreases cortical excitability (Huang, Edwards, Rounis, Bhatia, & Rothwell, 2005). Except for the direct impact on the cortical excitability of the stimulated site, rTMS can also influence the excitability of other brain regions related to the stimulation site probably through the cortico‐striato‐thalamocortical circuit (Gonzalez‐Garcia et al., 2011; Strafella, Paus, Barrett, & Dagher, 2001). Therefore, many studies have been undertaken to investigate the effectiveness of rTMS for PD patients with motor dysfunction.

Recent reviews Chou, Hickey, Sundman, Song, and Chen (2015), Zanjani, Zakzanis, Daskalakis, and Chen (2015) and Zhu et al. (2015) show the therapeutic effects of rTMS on motor dysfunction of PD patients as evaluated with the motor section of the Unified Parkinson's Disease Rating Scale (UPDRS‐III) and the optimal parameters of rTMS on the functional motor improvement of PD. Optimal parameters, however, have yet to be established. For example, many studies used rTMS protocols varying in stimulation parameters such as (a) stimulation sites of primary motor cortex (M1; Benninger et al., 2012; Bornke, Schulte, Przuntek, & Muller, 2004; Brys et al., 2016; Filipovic, Rothwell, van de Warrenburg, & Bhatia, 2009; Flamez et al., 2016; Khedr, Farweez, & Islam, 2003; Khedr, Rothwell, Shawky, Ahmed, & Hamdy, 2006; Kim et al., 2015; Lefaucheur et al., 2004; Maruo et al., 2013; Okabe, Ugawa, & Kanazawa, 2003; Siebner, Rossmeier, Mentschel, Peinemann, & Conrad, 2000; Yokoe et al., 2018) supplementary motor area (SMA; Brusa et al., 2006; Eggers, Günther, Rothwell, Timmermann, & Ruge, 2015; Hamada, Ugawa, & Tsuji, 2008; Koch et al., 2005; Shirota, Ohtsu, Hamada, Enomoto, & Ugawa, 2013; Yokoe et al., 2018) dorsal lateral prefrontal cortex (DLPFC; Brys et al., 2016; del Olmo, Bello, & Cudeiro, 2007; Nardone et al., 2014; Sedlackova, Rektorova, Srovnalova, & Rektor, 2009; Yokoe et al., 2018) the dorsal premotor (PMD; Sedlackova et al., 2009) and M1+ DLPFC (The M1+ DLPFC stimulation was considered as complex region stimulation; Benninger et al., 2011; Brys et al., 2016; Lomarev et al., 2006;) (b) LF‐rTMS (from 0.2 Hz to 1.0 Hz; Brusa et al., 2006; Filipovic et al., 2009; Flamez et al., 2016; Koch et al., 2005; Lefaucheur et al., 2004; Nardone et al., 2014; Okabe et al., 2003; Shirota et al., 2013) or HF‐rTMS (5.0 Hz: Hamada et al., 2008; Khedr et al., 2003; Koch et al., 2005; Siebner et al., 2000, 10.0 Hz: Bornke et al., 2004; Brys et al., 2016; del Olmo et al., 2007; Khedr et al., 2006; Kim et al., 2015; Lefaucheur et al., 2004; Maruo et al., 2013; Shirota et al., 2013; Sedlackova et al., 2009; Yokoe et al., 2018, 25.0 Hz: Lomarev et al., 2006 and 50.0 Hz: Benninger et al., 2012); (c) rTMS sessions: (one session: Bornke et al., 2004; Brusa et al., 2006; Eggers et al., 2015; Flamez et al., 2016; Koch et al., 2005; Lefaucheur et al., 2004; Nardone et al., 2014; Sedlackova et al., 2009; Siebner et al., 2000, three‐six sessions: Filipovic et al., 2009; Khedr et al., 2006; Kim et al., 2015; Maruo et al., 2013; Yokoe et al., 2018, eight sessions: Benninger et al., 2011; Benninger et al., 2012; Hamada et al., 2008; Lomarev et al., 2006; Okabe et al., 2003; Shirota et al., 2013; and 10 sessions: Brys et al., 2016; del Olmo et al., 2007; Flamez et al., 2016; Khedr et al., 2003;) (d) dosage of stimulation pulses (from 100 to 4,000 pulses/session); (e) medication status during assessment (“on” or “off” state); (f) the frequency of treatments (1–5 sessions/week); (g) the intensity above motor threshold (80%–120%MT); and (h) type of coil (Figure of 8 or circular coil). Often, rTMS of the M1 resulted in significant improvement of motor function, while rTMS over the SMA, DLPFC, and other sites produced less or no effects. Thus, different parameters between different rTMS studies have made it difficult to interpret and select the optimal rTMS stimulation parameters for PD motor improvement.

Additionally, previous reviews only assessed the rTMS studies that exclusively targeted at primary motor cortex (M1) or at different cortical areas (Elahi & Chen, 2009; Fregni, Simon, Wu, & Pascual‐Leone, 2005; Zanjani et al., 2015). Because the therapeutic effects of rTMS tended to be region‐specific (Kim, Kim, Chun, Yi, & Kwon, 2010; Levkovitz et al., 2015; Sasaki, Kakuda, & Abo, 2014) and dependent on stimulation frequency (Elahi & Chen, 2009) as well as sessions and pulses, (Chung & Mak, 2016) the aim of this meta‐analysis was to investigate the rTMS stimulation parameters that produced the optimal therapeutic effects on motor dysfunction of PD.

## 2 MATERIALS AND METHODS

### 2.1 Literature search

Multiple databases, which included PubMed, Medline, Embase, Science Direct, and the Cochrane library, were searched for relevant clinical studies published before 11 April 2018. The search terms were (“Parkinson Disease” or “Parkinson's Disease” or “Parkinsonism” or “Parkinsonian”) and (“repetitive transcranial magnetic stimulation” or “rTMS” or “repetitive TMS” or “theta burst stimulation” or “TBS”).

### 2.2 Inclusion criteria and exclusion criteria

The studies were included if they met the following criteria: (a) studies that evaluated the effectiveness of rTMS in adult patients with PD; (b) the design of studies was randomized controlled or crossover design; (c) studies that measured outcomes of motor dysfunction symptoms (motor examination of the Unified Parkinson's Disease Rating Scale [UPDRS‐III]); (d) the outcomes were reported or could be calculated from the original data of the study; and (e) the studies were published in English. Studies were excluded if the outcome assessments were not or could not be expressed as a mean value ± standard deviation (*SD*).

### 2.3 Study quality assessment

The methodological quality of included studies was assessed by two independent reviewers according to a slight modified checklist from Moher, Schulz, and Altman (2001) that contained six aspects: (1) randomization: recorded as “1” if the subjects were randomly allocated in the study and “0” if not; (2) blind procedure: “0” represented a nonblind or nondescribed procedure, and “1” and “2” represented single‐blind and double‐blind procedures, respectively; (3) dropout number was listed and recorded as the number of patients who dropped out from the study; (4) descriptions of baseline demographic and clinical characteristics were recorded as “1” if described, and “0” if not; (5) control design: recorded as “1” if the experiment was designed with healthy controls, “2” with patient controls, and “3” with both controls; and (6) adverse effects were denoted as a number of events.

### 2.4 Data extraction

The data from each study were extracted by two reviewers independently, and it included study design, sample size, sample characteristics, treatment parameters, medication status during assessment (“on” or “off”), outcome measurements, and the duration of the follow‐up (i.e., assessment made at 1 month or more after the last rTMS session was considered as long‐term outcome; Chung & Mak, 2016).

If the mean and *SD* of change scores were shown in the articles, they were extracted. If the mean and *SD* of the change scores were not clearly described in article, the change scores were calculated by using the following formula based on the principles of the Cochrane Handbook for Systematic Reviews of Interventions (Higgins, 2011):

$${\text{Mean}}_{\mathrm{change}}={\text{Mean}}_{\mathrm{final}}-{\text{Mean}}_{\mathrm{baseline}};$$

$$SD_{\mathrm{change}}=\sqrt{SD_{\mathrm{baseline}}^{2}+SD_{\mathrm{final}}^{2}-\left(2\times \text{Corr}\times SD_{\mathrm{baseline}}\times SD_{\mathrm{final}}\right)}$$

If the studies provided the mean values and the standard errors of the means, or reported original materials of each patient, then we calculated the mean values and *SD*s based on the principles of the Cochrane Handbook for Systematic Reviews of Interventions.Higgins, 2011 If the outcome was reported only as a graph, data were extracted by using the software GetData Graph Digitizer 2.25 (http://getdata-graph-digitizer.com/).

### 2.5 Data analysis

The data syntheses were conducted by using Reviewer Manager Version 5.3 software from the Cochrane Collaboration (Cochrane Collaboration, Oxford, England). The standardized mean difference (SMD) was calculated to estimate the effect size for clinical scores of the rTMS effect on motor symptoms measured with the UPDRS‐III, with 95% confidence interval (CI). The heterogeneity was tested by using the Cochran's Q statistics and *I* ^2^ test (Zintzaras & Ioannidis, 2005). If the *I* ^2^ value was greater than 50%, the random effect model was used for the analysis. Otherwise a fixed model was used. The Egger's test (Egger, Davey Smith, Schneider, & Minder, 1997) was used to test potential publication bias. Subgroup analysis was also performed based on the rTMS site, rTMS frequency, interaction between rTMS site and rTMS frequency, medication state during assessment (“on” or “off”), treatment sessions, and stimulation pulses. The statistical significance was set at *p* = 0.05.

## 3 RESULTS

### 3.1 Study selection

**Figure 1:**
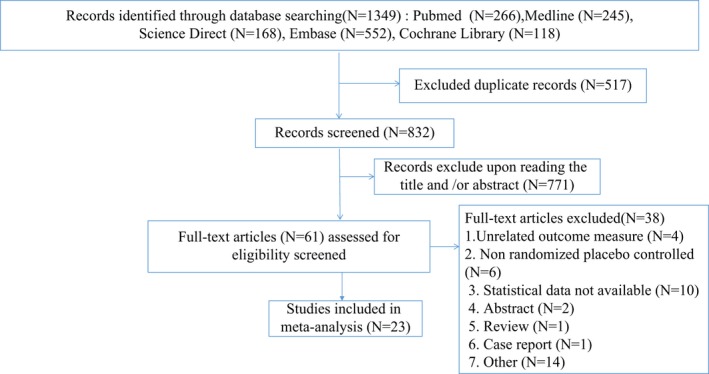
Flow diagram of literature search

Of a total of 1,349 relevant studies were found from the above‐mentioned databases, but only 23 studies were included in this meta‐analysis based on the inclusion and exclusion criteria. The flow diagram of the selection process is shown in Figure 1.

### 3.2 Quality assessment of the studies

Table 1 shows the quality assessments of the included studies. Randomized allocation of the patients was applied in most studies. Most of these studies were double‐blind or single‐blind. There was insufficient information to categorize the blind procedure in two studies (del Olmo et al., 2007; Sedlackova et al., 2009). Nine studies (Benninger et al., 2011, 2012; Brys et al., 2016; Hamada et al., 2008; Khedr et al., 2003; Kim et al., 2015; Lomarev et al., 2006; Sedlackova et al., 2009; Shirota et al., 2013) described the dropout number.

**Table 1:** Quality assessment of the included studies

Study	Randomization	Blind procedure	Control design	Descriptions of baseline demographic and clinical characteristics	Dropout number	Adverse effects
Lefaucheur et al. (2004)	1	2	2	1	0	0
Filipovic et al. (2009)	1	1	2	1	0	0
Flamez et al. (2016)	1	2	2	1	0	0
Siebner et al. (2000)	1	1	2	1	0	0
Khedr et al. (2003)	1	2	2	1	5	0
Bornke et al. (2004)	1	2	2	1	0	0
Maruo et al. (2013)	1	2	2	1	0	0
Khedr et al. (2006)	1	2	2	1	0	U
Benninger et al. (2012)	1	2	2	1	1	0
Brys et al. (2016)	1	2	2	1	11	34
Okabe et al. (2003)	1	2	2	1	0	NR
Shirota et al. (2013)	1	2	2	1	11	2
Brusa et al. (2006)	1	2	2	1	0	0
Koch et al. (2005)	1	2	2	1	0	NR
Hamada et al. (2008)	1	2	2	1	5	NR
del Olmo et al. (2007)	1	0	2	1	0	NR
Sedlackova et al. (2009)	1	0	2	1	1	NR
Nardone et al. (2014)	1	1	3	1	0	0
Lomarev et al. (2006)	1	2	2	1	2	1
Kim et al. (2015)	1	2	2	1	2	1
Benninger et al. (2011)	1	2	2	1	2	9
Eggers et al. (2015)	0	1	2	1	0	0
Yokoe et al. (2018)	1	2	2	1	0	1

Note

U: unclear, insufficient information to count the number of adverse events; NR: not reported.

### 3.3 Study characteristics

The main characteristics of the studies are summarized in Tables 2 and 3. Table 2 includes the study design, sample size, and the main characteristics of the subjects. Of the 23 studies selected for this meta‐analysis, 10 studies (Benninger et al., 2011; del Olmo et al., 2007; Hamada et al., 2008; Khedr et al., 2003, 2006; Lomarev et al., 2006; Okabe et al., 2003; Shirota et al., 2013) were parallel controlled and 13 studies (Bornke et al., 2004; Brusa et al., 2006; Eggers et al., 2015; Filipovic et al., 2009; Flamez et al., 2016; Kim et al., 2015; Koch et al., 2005; Lefaucheur et al., 2004; Maruo et al., 2013; Nardone et al., 2014; Sedlackova et al., 2009; Siebner et al., 2000; Yokoe et al., 2018) were crossover controlled. Table 3 shows the rTMS stimulation parameters including the site, frequency, intensity, sessions.

**Table 2:** Characteristics of the included trails

Study	Design	Sample size	Age(y)	Sex (M/F)	Disease duration (y)	H&Y stage
Benninger et al. (2012)	Parallel	Ne:13 Nc:13	Ne:64.5 ± 9.1 Nc:63.7 ± 8.3	Ne:11/2 Nc:9/4	Ne:8.6 ± 4.1 Nc:9.3 ± 6.8	2–4
Khedr et al. (2003)	Parallel	Ne:19 Nc:17	Ne:57.8 ± 9.2 Nc:57.5 ± 8.4	Ne:14/5 Nc:10/7	Ne:3.05 ± 2.1 Nc:3.6 ± 2.4	2–3
Kim et al. (2015)	Crossover	17	64.5 ± 8.4	12/5	7.8 ± 4.9	3.0 ± 0.5
Khedr et al. (2006)	Parallel	Ne:10 Nc:10	Ne:60.2 ± 9.48 Nc:60.6 ± 10.3	NR	Ne:4.6 ± 2.26 Nc:4.6 ± 1.97	Ne:3.5 ± 0.7 Nc:3.8 ± 0.9
Bornke et al. (2004)	Crossover	12	55.92 ± 13.09	6/6	NR	2.25 ± 0.8
Filipovic et al. (2009)	Crossover	10	64.5 ± 9.6	5/5	15.6 ± 5.7	NR
Flamez et al. (2016)	Crossover	9	70 ± 5.9	4/5	13.8 ± 5.6	3 ± 1
	Crossover	6	68.8 ± 10.3	4/2	14.0 ± 5.0	3 ± 1
Lefaucheur et al. (2004)	Crossover	12	64 ± 6.9	7/5	11 ± 3.5	3.4 ± 0.2
Maruo et al. (2013)	Crossover	21	63.0 ± 11.3	11/10	12.0 ± 6.3	3.1 ± 0.5
Siebner et al. (2000)	Crossover	10	57 ± 11	7/3	5.5 ± 3.4	1–2.5
Hamada et al. (2008)	Parallel	Ne:55 Nc:43	Ne:65.3 ± 8.9 Nc:67.4 ± 8.5	Ne:29/24 Nc:25/15	Ne:8.1 ± 4.2 Nc:7.8 ± 6.7	2–4
Shirota et al. (2013)	Parallel	34/34/34	L:68.8 ± 67.6 H:67.9 ± 68.4 Nc:65.7 ± 68.5	NR	L:8.5 ± 7.3 H:7.8 ± 6.6 Nc:7.6 ± 4.4	2–4
Brusa et al. (2006)	Crossover	10	61 ± 8.04	6/4	16.4 ± 5.4	NR
Koch et al. (2005)	Crossover	8	60.75 ± 9.84	4/4	16.5 ± 5.93	NR
Nardone et al. (2014)	Crossover	4	65.75 ± 3.5	3/1	10.25 ± 4.57	NR
del Olmo et al. (2007)	Parallel	Ne:8 Nc:5	61.7 ± 5.22	6/7	8 ± 5	1–3
Sedlackova et al. (2009)	Crossover	10	63.7 ± 6.7	9/1	7.8 ± 6.5	NR
	Crossover	10	63.7 ± 6.7	9/1	7.8 ± 6.5	NR
Brys et al. (2016)	Parallel	Ne:14 Nc:15	Ne:59.6 ± 12.6 Nc:64.0 ± 7.4	Ne:9/5 Nc:11/4	Ne:8.4 ± 5.2 Nc:4.5 ± 2.2	2–4
	Parallel	Ne:12 Nc:15	Ne:64.6 ± 12.3 Nc:64.0 ± 7.4	Ne:6/6 Nc:11/4	Ne:7.7 ± 4.2 Nc:4.5 ± 2.2	2–4
	Parallel	Ne:20 Nc:15	Ne:64.9 ± 8.0 Nc:64.0 ± 7.4	Ne:11/9 Nc:11/4	Ne:7.3 ± 5.6 Nc:4.5 ± 2.2	2–4
Lomarev et al. (2006)	Parallel	Ne:9 Nc:9	Ne:63 ± 10 Nc:66 ± 10	Ne:7/2 Nc:8/1	Ne:13.8 ± 6.8 Nc:10.8 ± 3.1	2–4
Okabe et al. (2003)	Parallel	Ne:28 Nc:28	67.2 ± 8.2	NR	Ne:8.8 ± 5.1 Nc:8.0 ± 5.4	Ne:3.11 ± 0.92 Nc:2.92 ± 0.83
	Parallel	Ne:29 Nc:28	67.2 ± 8.2	NR	Ne:8.8 ± 6.4 Nc:8.0 ± 5.4	Ne:2.95 ± 0.83 Nc:2.92 ± 0.83
Benninger et al. (2011)	Parallel	Ne:13 Nc:13	Ne:62.1 ± 6.9 Nc:65.6 ± 9.0	Ne:7/6 Nc:11/2	Ne:10.8 ± 7.1 Nc:6.5 ± 3.4	Ne:2.6 ± 0.2(on) 3.0 ± 0.4(off) Nc:2.5 ± 0.1(on) 2.9 ± 0.2(off)
Eggers et al. (2015)	Crossover	13	Off:64.7 ± 5.0	Off:4/9	Off:5.8 ± 4.3	Off:1.8 ± 0.8
	Crossover	13	On:60.8 ± 7.8	On:6/7	On:7.1 ± 4.7	On:1.7 ± 0.8
Yokoe et al. (2018)	Crossover	19	69.1 ± 8.4	7/12	9.5 ± 3.2	3.5 ± 0.6
	Crossover	19	69.1 ± 8.4	7/12	9.5 ± 3.2	3.5 ± 0.6
	Crossover	19	69.1 ± 8.4	7/12	9.5 ± 3.2	3.5 ± 0.6

Note

H&Y, Hoehn and Yahr Stage; Ne, number of experimental group; Nc, number of control group; NR, not reported.

**Table 3:** Treatment parameters, clinical assessment, and follow‐up

Study	Stimulation parameters					Sham rTMS	Status	Session duration	Follow‐up
	Frequency	Intensity	Coil‐type	Sessions‐pulse	Site				
Benninger et al. (2012)	50 Hz	80%AMT	C	600	M1	Inactive and active coils	On and Off	8/2 weeks	1 month
Khedr et al. (2003)	5 Hz	120%RMT	F8	2,000	M1	Tilted coil	Off	10/10 day	1 month
Kim et al. (2015)	10 Hz	90%RMT	Dc	1,000	M1	Tilted coil	On	5/5 days	2 weeks
Khedr et al. (2006)	10 Hz	100%RMT	F8	3,000	M1	Occipital stimulation	Off	6/6 days	1 month
Bornke et al. (2004)	10 Hz	90%RMT	F8	1,000	M1	Tilted coil	Off	1 days	No
Filipovic et al. (2009)	1 Hz	~90%RMT	F8	1,800	M1	Sham coil	On and Off	4/4 days	No
Flamez et al. (2016)	1 Hz	90%RMT	F8	2,000	M1	Tilted coil	On	1 day	No
	1 Hz	90%RMT	F8	2,000	M1	Tilted coil	On	10/5 days	2 weeks
Lefaucheur et al. (2004)	L:0.5 Hz	80%RMT	F8	L:600	M1	Sham coil	Off	1 day	No
	H:10 Hz	80%RMT	F8	H:2,000	M1	Sham coil	Off	1d	No
Maruo et al. (2013)	10 Hz	100%RMT	F8	1,000	M1	Realistic Sham	Off	3/3 days	No
Siebner et al. (2000)	5 Hz	90%RMT	F8	2,250	M1	Tilted coil	Off	1 day	No
Hamada et al. (2008);	5 Hz	110%AMT	F8	1,000	SMA	Realistic Sham	On	8/8 weeks	1 month
Shirota et al. (2013)	L:1 Hz	110%AMT	F8	L:1,000	SMA	Realistic Sham	On	8/8 weeks	1, 12 weeks
	H:10 Hz	110%AMT	F8	H:1000	SMA	Realistic Sham	On	8/8 weeks	1, 12 weeks
Brusa et al. (2006)	1 Hz	90%RMT	F8	900	SMA	Tilted coil	On	1 day	No
Koch et al. (2005)	L:1 Hz	90%RMT	F8	900	SMA	Tilted coil	On	1 day	No
	H:5 Hz	110%RMT	F8	900	SMA	Tilted coil	On	1 day	No
Nardone et al. (2014)	1 Hz	Below AMT	F8	1800	DLPFC	Sham coil	On	1 day	No
del Olmo et al. (2007)	10 Hz	90%AMT	F8	450	DLPFC	Tilted coil	On	10/10 days	No
Sedlackova et al. (2009)	10 Hz	100%RMT	F8	1350	DLPFC	Occipital Stimution	Off	1 day	No
	10 Hz	100%RMT	F8	1,350	PMD	Occipital Stimution	Off	1 day	No
Brys et al. (2016)	10 Hz	NR	F8	2,000	M1	Sham coil	Off	10/2 weeks	1 week, 1,3,6 months
	10 Hz	NR	F8	2,000	DLPFC	Sham coil	Off	10/2 weeks	1,3,6 months
	10 Hz	NR	F8	3,000	M1 + DLPFC	Sham coil	Off	10/2 weeks	1,3,6 months
Lomarev et al. (2006)	25 Hz	100%RMT	F8	1,200	M1 + DLPFC	Coil inactive surface	On and off	8/4 weeks	1 months
Okabe et al. (2003)	0.2 Hz	110%AMT	C	100	M1	Sham coil	On	8/8 weeks	1,2 months
	0.2 Hz	110%AMT	C	100	Occipital	Sham coil	On	8/8 weeks	1,2 months
Benninger et al. (2011)	50 Hz	80%AMT	C	2,400	M1 + DLPFC	Sham coil	On and off	8/2 weeks	1 months
Eggers et al. (2015)	50 Hz	90%AMT	F8	600	SMA	Title coil	Off	1 day	No
	50 Hz	90%AMT	F8	600	SMA	Title coil	On	1 day	No
Yokoe et al. (2018)	10 Hz	100%RMT	C	1,000	M1	Sham coil	On	3 days	No
	10 Hz	100%RMT	C	1,000	SMA	Sham coil	On	3 days	No
	10 Hz	100%RMT	C	1,000	DLPFC	Sham coil	On	3 days	No

Note

AMT: active motor threshold; RMT: resting motor threshold; C: circular; F8: figure of 8; Dc: double coil; M1: primary motor cortex; SMA: supplementary motor area; DLPFC: dorsolateral prefrontal cortex; PMD: dorsal premotor cortex; NR: not reported; H: high‐frequency group; L: low‐frequency group.

### 3.4 Adverse effects

Seventeen studies (Benninger et al., 2011, 2012; Bornke et al., 2004; Brusa et al., 2006; Brys et al., 2016; Filipovic et al., 2009; Flamez et al., 2016; Khedr et al., 2003, 2006; Kim et al., 2015; Lefaucheur et al., 2004; Lomarev et al., 2006; Maruo et al., 2013; Nardone et al., 2014; Shirota et al., 2013; Siebner et al., 2000; Yokoe et al., 2018) evaluated the incidence of adverse effects. Of these, 11 studies (Benninger et al., 2012; Bornke et al., 2004; Brusa et al., 2006; Eggers et al., 2015; Filipovic et al., 2009; Flamez et al., 2016; Khedr et al., 2003; Lefaucheur et al., 2004; Maruo et al., 2013; Nardone et al., 2014; Siebner et al., 2000) showed no adverse effects of rTMS. Khedr et al. (2006) reported an occasional mild, transient headache in some patients. Brys et al. (2016) reported adverse effects in 25 active rTMS and nine sham rTMS‐treated PD patients, respectively. The most common adverse effects were mild and transient headache and neck pain. Shirota et al. (2013) reported that two patients experienced tinnitus and headache. Kim et al. (2015) reported that one patient had a mild headache which disappeared soon after the stop of rTMS. Lomarev et al. (2006) reported that one patient could not tolerate the pain under the coil in the rTMS group. Benninger et al. (2011) reported that nine patients had occasional local pain or discomfort during iTBS stimulation, predominantly of DLPFC, and one patient had an isolated, nonpulsatile, left‐sided tinnitus for a few minutes. Yokoe et al. (2018) reported that one patient had a mild headache during the DLPFC stimulation that spontaneously resolved.

### 3.5 Synthesis of results

**Figure 2:**
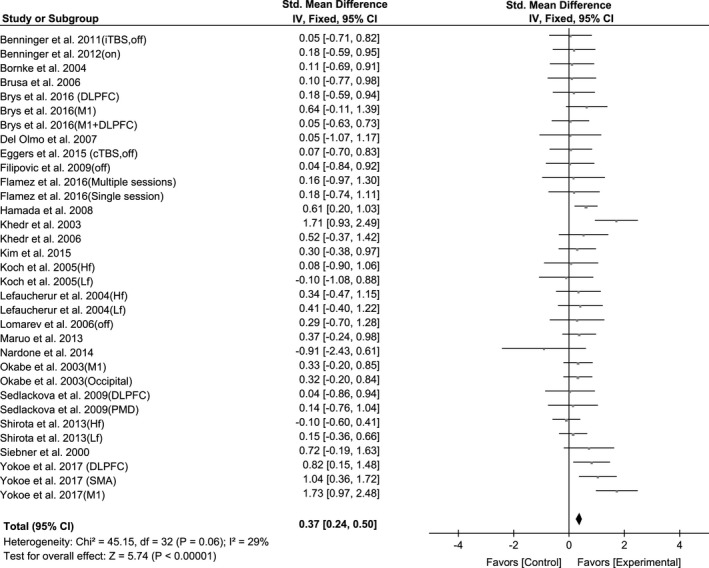
Forest plot from the meta‐analysis of rTMS on UPDRS‐III scores at short‐term showing estimates of mean effect size with 95% confidence interval (95% CI). Studies denoted as “on” or “off” distinguish those with assessment done both in “on” and “off” medication state

A total of 33 trials of the selected 23 articles compared the effect of rTMS on motor signs. The results of pooled data showed a significant improvement after rTMS therapy (SMD, 0.37; 95% CI, 0.24–0.50; *p* < 0.00001; *I* ^2^ = 29%; Figure 2). The Egger's test (*p* = 0.41) showed no significant publication bias.

**Figure 3:**
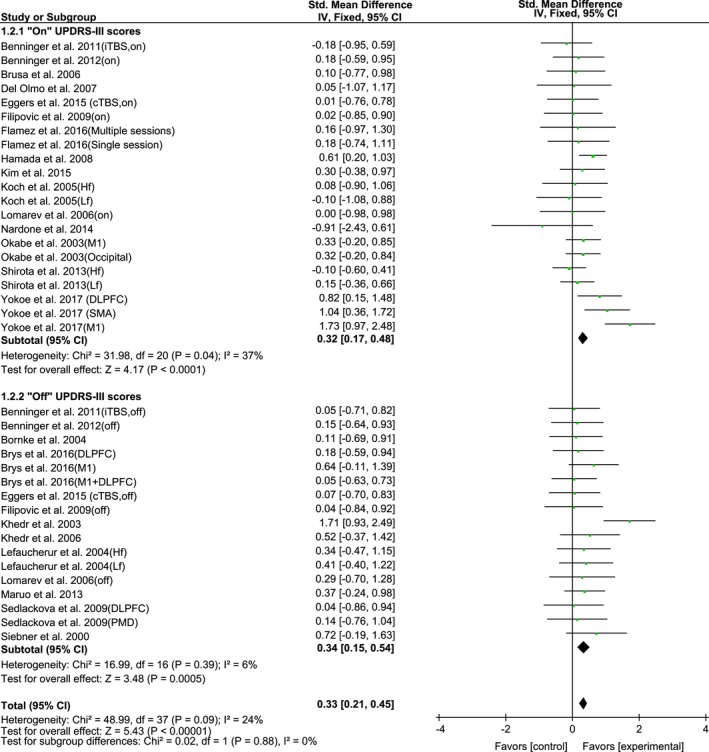
Forest plot of rTMS on UPDRS‐III scores measured during “on” and “off” medication state showing estimates of mean effect size with 95% confidence interval (95% CI). Studies denoted as “on” or “off” distinguish those with assessment done both in “on” and “off” medication state

The result of the subgroup analysis for medication state (“on” or “off”) during assessment was as also calculated (Figure 3). No significant difference of the mean therapeutic effect size of rTMS for UPDRS‐III was observed between “on” state (SMD, 0.32; 95% CI, 0.17–0.48; *p* < 0.0001; *I* ^2^ = 37%) and “off” state (SMD, 0.34; 95% CI, 0.15–0.54; *p* = 0.0005; *I* ^2^ = 6%).

**Figure 4:**
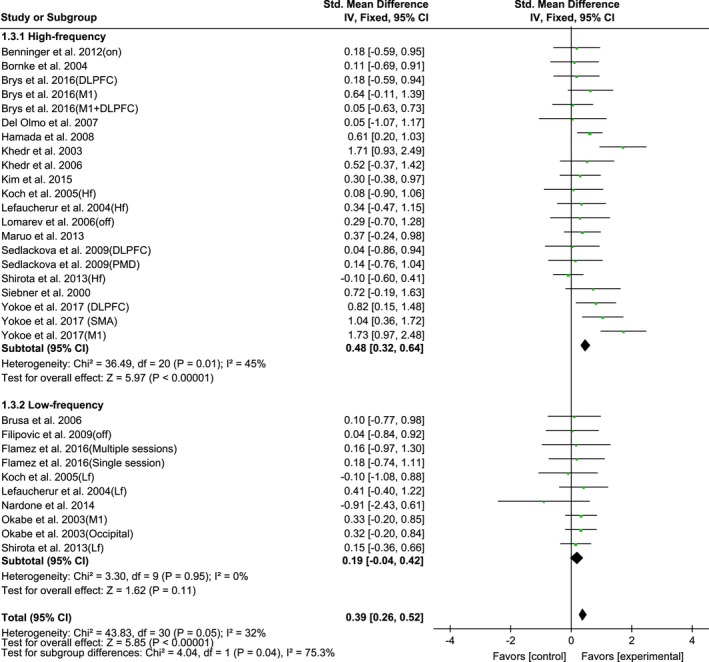
Forest plots of rTMS on UPDRS‐III scores for studies comparing high‐frequency and low‐frequency rTMS protocol showing estimates of mean effect size with 95% confidence interval (95% CI). Studies denoted as “on” or “off” distinguish those with assessment done both in “on” and “off” medication state

The stimulation frequency subgroup analysis is presented as Figure 4. The frequency subgroup analysis revealed a significant improvement in the UPDRS‐III only after HF‐rTMS (SMD, 0.48; 95% CI, 0.32–0.64; *p* < 0.00001; *I* ^2^ = 45%) but not after LF‐rTMS (SMD, 0.19; 95% CI, −0.04–0.42; *p* = 0.11; *I* ^2^ = 0%).

**Figure 5:**
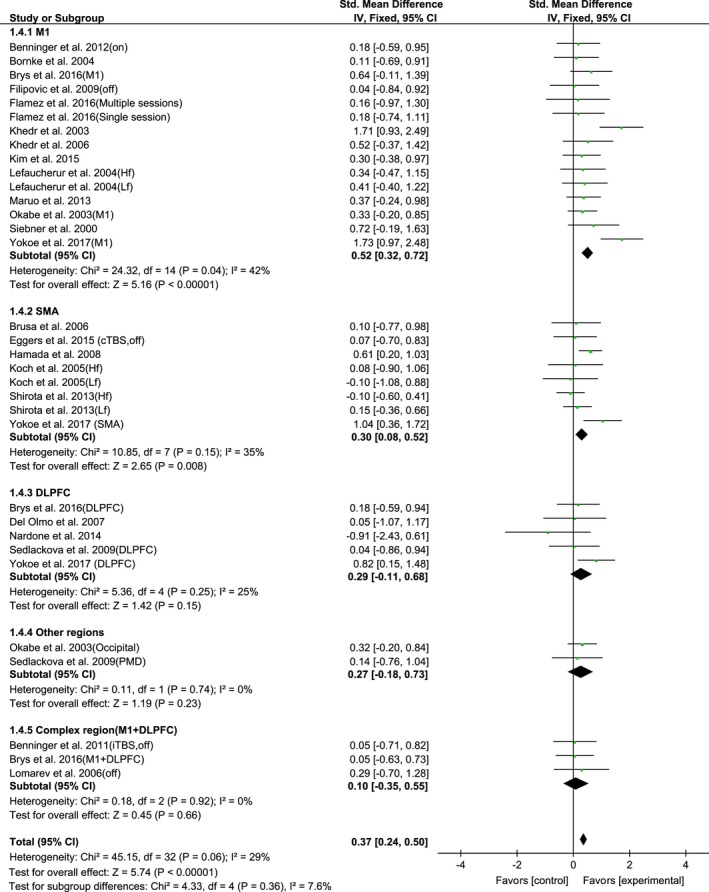
Forest plots of rTMS on UPDRS‐III scores for studies comparing the stimulation site of primary motor cortex (M1), supplementary motor area (SMA), dorsal lateral prefrontal cortex (DLPFC), the other regions and complex region (M1 +  DLPFC) showing estimates of mean effect size with 95% confidence interval (95% CI). Studies denoted as “on” or “off” distinguish those with assessment done both in “on” and “off” medication state

The subgroup analysis of rTMS sites showed the following order of mean effect sizes of rTMS on UPDRS‐III: 0.52 (95% CI, 0.32–0.72; *p* < 0.00001; *I* ^2^ = 42%) for M1; 0.30 (95% CI, 0.08–0.52; *p* = 0.008; *I* ^2^ = 35%) for SMA; 0.29 (95% CI, −0.11–0.68; *p* = 0.15; *I* ^2^ = 25%) for DLPFC; 0.27 (95% CI, −0.18–0.73; *p* = 0.23; *I* ^2^ = 0%) for other regions, and 0.10 (95% CI, −0.35–0.55; *p* = 0.66; *I* ^2^ = 0%) for complex region, respectively (Figure 5). The M1 and SMA sites revealed the significant improvement after rTMS therapy.

**Figure 6:**
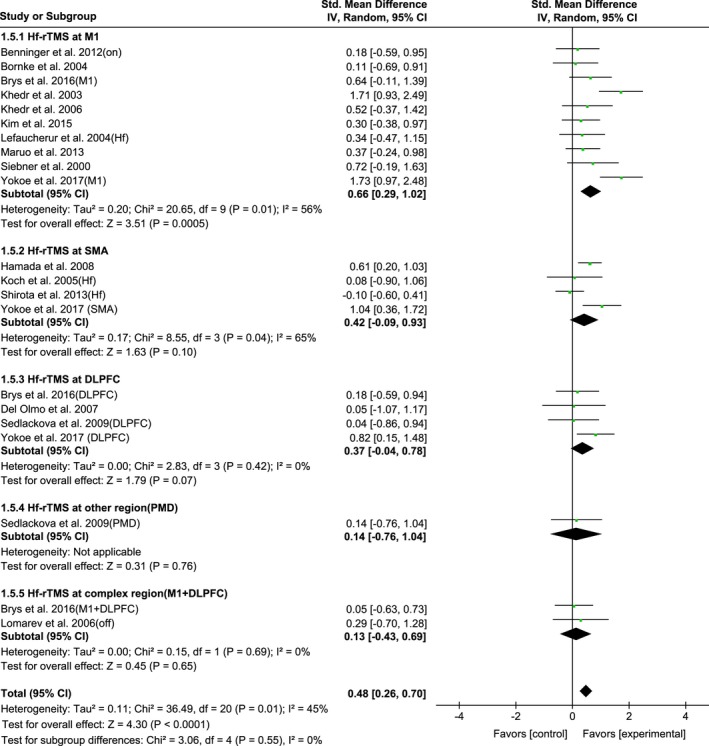
The interaction between high‐frequency rTMS and stimulation site of rTMS on UPDRS‐III scores showing estimates of mean effect size with 95% confidence interval (95% CI). Studies denoted as “on” or “off” distinguish those with assessment done both in “on” and “off” medication state

As the HF‐rTMS was more effective than LF‐rTMS for improving Parkinson's motor symptoms (Figure 4), we further re‐analyzed the site effect of HF‐rTMS therapy on UPDRS‐III, with the following results: 0.66 (95% CI, 0.29–1.02; *p* = 0.0005; *I* ^2^ = 56%) for M1; 0.42 (95% CI, −0.09–0.93; *p* = 0.1; *I* ^2^ = 65%) for SMA; 0.37 (95% CI, −0.04–0.78; *p* = 0.07; *I* ^2^ = 0%) for DLPFC; 0.14 (95% CI, −0.76–1.04; *p* = 0.76) for other region, and 0.13 (95% CI, −0.43–0.69; *p* = 0.65; *I* ^2^ = 0%) for complex region (M1 + DLPFC), respectively (Figure 6).

**Figure 7:**
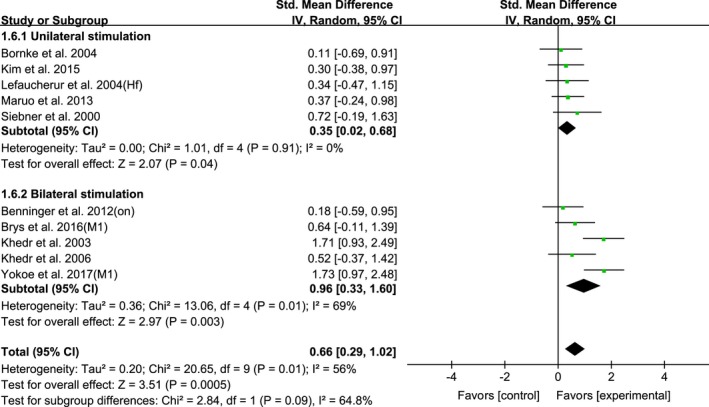
Forest plots of HF‐rTMS over M1 on UPDRS‐III scores for studies comparing the bilateral vs unilateral stimulation of rTMS protocol showing estimates of mean effect size with 95% confidence interval (95% CI). Studies denoted as “on” or “off” distinguish those with assessment done both in “on” and “off” medication state

The bilateral/unilateral stimulation would also influence the rTMS effect. The mean effect sizes of bilateral and unilateral stimulation of HF‐rTMS over M1 were the following results: 0.96 (95% CI, 0.33–1.60; *p* = 0.003; *I* ^2^ = 69%) and 0.35 (95% CI, 0.02–0.68; *p* = 0.04; *I* ^2^ = 0%), respectively (Figure 7).

**Figure 8:**
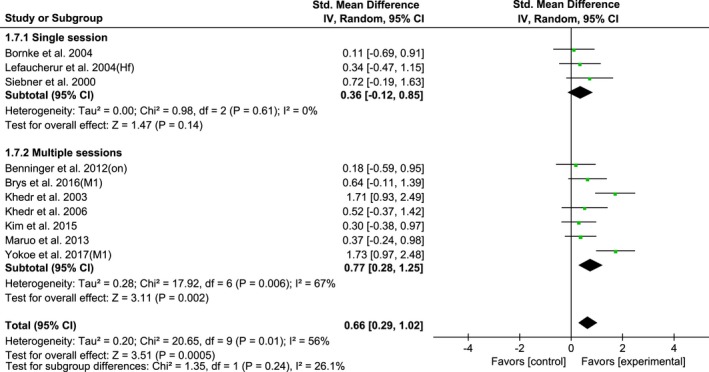
Forest plots of HF‐rTMS over M1 on UPDRS‐III scores for studies comparing the single‐ and multi‐session of rTMS protocol showing estimates of mean effect size with 95% confidence interval (95% CI). Studies denoted as “on” or “off” distinguish those with assessment done both in “on” and “off” medication state

Ten studies of HF‐rTMS over M1 were further divided into subgroups according to the single‐session and multi‐session of rTMS treatment: single‐session (SMD, 0.36; 95% CI, −0.12–0.85; *p* = 0.14; *I* ^2^ = 0%) and multi‐session (SMD, 0.77; 95% CI, 0.28–1.25; *p* = 0.002; *I* ^2^ = 67%) (Figure 8). Multi‐session showed a significantly larger effect size than single‐session rTMS.

**Figure 9:**
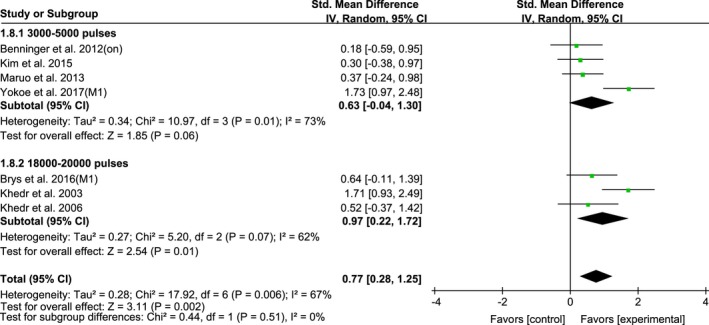
Forest plots of HF‐rTMS over M1 on UPDRS‐III scores for studies comparing the different total pulses of rTMS protocol showing estimates of mean effect size with 95% confidence interval (95% CI). Studies denoted as “on” or “off” distinguish those with assessment done both in “on” and “off” medication state

The mean effect sizes of the multi‐session total numbers of HF‐rTMS pulses (3,000–5,000 pulses and 18,000–20,000 pulses) delivered over M1 were the following results: 0.63 (95% CI, −0.04–1.3; *p* = 0.06; *I* ^2^ = 73%) and 0.97 (95% CI, 0.22–1.72; *p* = 0.01; *I* ^2^ = 62%) (Figure 9).

### 3.6 Long‐term follow‐up of motor outcome

**Figure 10:**
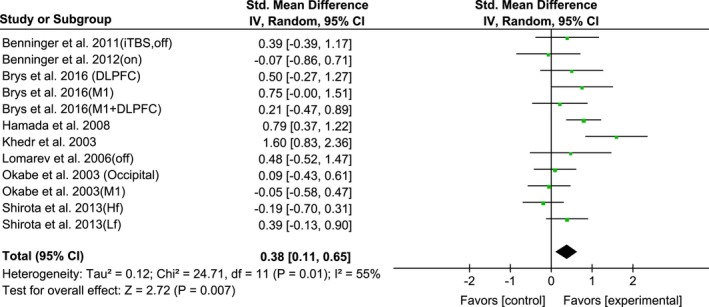
Forest plot from the meta‐analysis of rTMS on UPDRS‐III scores at long‐term estimates of mean effect size with 95% confidence interval (95% CI). Studies denoted as “on” or “off” distinguish those with assessment done both in “on” and “off” medication state

Twelve trials reported the long‐term effect size of rTMS for UPDRS‐III. The pooled effect size showed significant benefit effect of rTMS (SMD, 0.38; 95% CI, 0.11–0.65; *p* = 0.007) with a moderate level of heterogeneity (*I* ^2^ = 55%) and a lack of any publication bias (Egger's test, *p* = 0.57) (Figure 10).

## 4 DISCUSSION

This meta‐analysis indicated more accurate evidence to support the efficacy of rTMS on motor recovery of PD patients. These results suggest that rTMS might be helpful in improving the motor deficits of PD patients. In particular, HF‐rTMS appears more significant effect than LF‐rTMS and significant difference also is found; stimulation over M1 shows better efficacy than other stimulation sites; bilateral stimulation is more effective than unilateral stimulation; multi‐session and higher dosage of pulses (total 18,000–20,000 pulses) are associated with better motor outcome. Additionally, the effect of rTMS appears to be long‐lasting and unaffected by the patient's medication state.

Subgroup analysis result for the effects of HF‐rTMS versus LF‐rTMS was consistent with the previous report of Elahi and Chen (2009). Who found a significant effect size of UPDRS‐III for HF‐rTMS studies but not significant for LF‐rTMS studies. Distinct results, however, were observed in some other meta‐analysis studies in which no significant difference between HF‐rTMS and LF‐rTMS was found (Chou et al., 2015); an opposite result was discovered (Wagle Shukla et al., 2016) or no significant result was observed both in HF‐rTMS and LF‐rTMS (Chung & Mak, 2016). The reasons for these distinctions may be due to the differences in the included trials, (Zhu et al., 2015) data extraction, (Chou et al., 2015) and statistical methods (Wagle Shukla et al., 2016). Compared with these previous published meta‐analyses, this study enrolled more trials and calculated more detailed subgroup analyses. Therefore, except for the consistent results with previous studies, more new results were discovered including bilateral stimulation, HF‐rTMS over M1 in this meta‐analysis.

The subgroup analysis for rTMS site in this current analysis showed that rTMS targeting the M1 and SMA were significantly effective for PD patients with motor signs. In addition, rTMS over the M1 is more effective than stimulation over the SMA. A previous study gained the similar result (Chung & Mak, 2016). Moreover, Zanjani et al. (2015) only included the studies that rTMS targeted the M1 which also showed positive effect. However, the combination of rTMS frequency and rTMS site in this current analysis revealed that only HF‐rTMS at M1 showed significant effect size, HF‐rTMS targeting the SMA, DLPFC, and M1 + DLPFC were insignificant. The neuroimaging studies which use simple motor tasks to investigate bradykinesia‐related neural activity in PD patients showed significantly decreased activation in the caudate nucleus among other regions (Rolls, Thorpe, & Maddison, 1983). It is known that PD results from the loss of dopaminergic innervation to the dorsal striatum, a main components of the basal ganglia that is highly innervated by dopamine neurons that originate from the substantia nigra pars compacta (SNc). Basal ganglia are also strongly interconnected with the cerebral cortex (including the M1, thalamus, brainstem, and several other brain areas).

A hypo active caudate nucleus may underlie the motor deficits in PD patients through interfering with the normal functioning of the striato‐frontal motor loop (Rolls et al., 1983). Gonzalez‐Garcia et al. (2011) found that HF‐rTMS over the M1 significantly improved motor behavior and increased motor‐related activity in the caudate nucleus. It has been suggested that striatal dopamine depletion results in an over‐inhibition of neuronal activity in the motor thalamus and consequently the hypo activity in cortical motor and association areas (Conditions TNCCfC, 2006). Other study showed that rTMS applied to the M1 increased dopamine release in the nigrostriatal system (Strafella, Paus, Fraraccio, & Dagher, 2003). Therefore, HF‐rTMS over the M1 may improve motor function through increased motor‐related activity in the caudate nucleus and dopamine release in the nigrostriatal system.

Previous studies indicate that HF‐rTMS could facilitate SMA activation and provide symptomatic relief in PD patients (Jenkins et al., 1992; Playford et al., 1992; Rascol et al., 1997). Hamada et al. (2008) and Yokoe et al. (2018) reported prominent motor improvement after HF‐rTMS over the SMA. However, that result was not reproduced by a multicenter trial with the same stimulation site and session numbers (Shirota et al., 2013). Two other studies showed no effect of one session HF‐rTMS (Koch et al., 2005) or LF‐rTMS (Brusa et al., 2006) over the SMA on UPDRS‐III. More studies are needed to clarify the effectiveness of rTMS targeting the SMA.

Despite of the cortico‐cortical connections with the parietal and premotor cortices’ involvement in visuo‐motor control of action and the crucial role of DLPFC in the cognitive control of motor behavior (Kim et al., 2015), our meta‐analysis did not show significant motor benefit after rTMS over the DLPFC in terms of the UPDRS‐III scores. Other studies showed no effect of rTMS over DLPFC on clinical Parkinsonian symptoms, motor performance of ballistic wrist movements,or synergistic effects of motor signs improvement of rTMS over DLPFC+M1 (Conditions TNCCfC, 2006). However, it has been suggested that changes in different motor tasks specifically related to DLPFC function (motor planning and response selection) could be more sensitive to measure motor improvement induced by rTMS over this area (Kim et al., 2015).

The subgroup analysis for bilateral versus unilateral stimulation of HF‐rTMS over the M1 showed that the bilateral stimulation over the M1 was more effective than unilateral stimulation over the M1 for PD with motor signs. The greater motor improvement in bilateral stimulation suggests that bilateral M1 stimulation may have a superimposed effect.

The efficacy of HF‐rTMS appears to be session number‐dependent. Our results showed that, in the short‐term, multi‐session of HF‐rTMS over the M1 is more effective than a single‐session in improving the motor deficits. Khedr et al. (2003) showed gradual development of long‐lasting effects over repeated sessions of rTMS. Repeated and long‐term TMS of the brain may cause lasting effects on excitability probably due to cumulative effects of multi‐session on synaptic connections analogous to long‐term potentiation (Platz & Rothwell, 2010). More sessions of rTMS are often associated with increased dosage or numbers of rTMS stimulation pulses which is another factor underlying the efficacy of rTMS. It would be interesting to know if there are any differences in efficacy between a single‐session and multiple sessions of rTMS that delivered equal doses of pulses. That could reveal the optimal combination of session numbers and pulse dosage for best therapeutic outcomes. In this analysis, we had few single‐session trials that had different doses of pulses to allow such analysis. Our subgroup analysis showed the effect size was statistically significant for trials of multiple sessions with higher number of total stimulation pulses (range from 18,000 to 20,000 pulses). This is in line with an earlier review that indicates a larger effect size of rTMS on UPDRS‐III scores is associated with a greater number of stimulation pulses (Chung & Mak, 2016). One study of rTMS on major depression showed that 1,200–1,500 pulses/day for 10 or 20 sessions (a total of dose between 12,000 and 30,000 pulses) had the best anxiolytic effects (Teng et al., 2017). Based on these results and others, one can anticipate that an optimal daily pulses dosage of HF‐rTMS over the M1 for an extended period of time could deliver an optimal efficacy for PD patients.

Previous studies evaluated the long‐term effect of rTMS on motor function in PD patients (Chou et al., 2015; Chung & Mak, 2016; Wagle Shukla et al., 2016; Zanjani et al., 2015). Chou et al. (2015) reported the significant long‐term improvement in motor function in PD patients, and the follow‐up was conducted at 1 week or more after the last rTMS session. However, the other two studies estimated the long‐term effects of rTMS when the follow‐up was conducted at 1 month or more after the last rTMS session (Chung & Mak, 2016; Zanjani et al., 2015). Zanjani et al. (2015) reported nonsignificant effect size while Chung and Mak (2016) reported significant long‐term effects. Wagle Shukla et al. (2016) reported nonsignificant long‐term effects, and in this study, there was no clear definition of how long after the last rTMS session was considered as long‐term follow‐up. They only reported the average follow‐up period, it was 6 weeks. Our present meta‐analysis showed the significant long‐term improvement in motor function in PD patients after rTMS treatment. In this meta‐analysis, the long‐term effect refers to the effect at 1 month or more after the last multiple rTMS sessions of treatment, while single‐session does not involve long‐term effects. The postsynaptic changes and the expansion of the short‐term range of plasticity may underlie the cumulative long‐lasting effect of rTMS in patients with PD (Lomarev et al., 2006). Repeated episodes of long‐term potentiation could also result in increased synapses activity and strength to facilitate neural remodeling (Leuner & Shors, 2004; Matsuzaki, Honkura, Ellis‐Davies, & Kasai, 2004). The long‐term effect in this meta‐analysis may be similar with the theory of Cohen et al. (2018) concerning maintenance treatment that discontinuous treatment will reduce the effect of treatment, the maintenance treatment is a routine treatment for electroshock treatment.

The differences in long‐term effects of these studies may be due to the following reasons. In this current analysis, Khedr et al. (2003) were included. In their study, the patients received no anti‐Parkinsonism medication for at least 1 month before the start of the study. This is different from the patients of other included studies. Also rTMS was applied for lower limbs and hand (right then left hemispheres) with the total numbers of 20,000 pulses. It produced greater effect size than most other studies. The result of this study may affect the reliability of the results in this analysis. However, when this study was removed, our meta‐analysis still showed significant results.

There are still limitations of this meta‐analysis. First, the experimental designs of the included studies were not homogenous (e.g., randomized controlled trials versus crossover design). Second, the selected participants varied in age, disease stage, and other biological characteristics that may have confounded the results, and third, non‐English studies were excluded in this meta‐analysis.

In conclusion, this meta‐analysis shows significant benefits of HF‐rTMS therapy over the M1 on motor signs of PD patients as measured by UPDRS‐III. Multi‐sessions of HF‐rTMS over the M1 (especially bilateral M1) with a total of 18,000–20,000 pulses appear to have produced the better efficacy.

## CONFLICTS OF INTEREST

The authors declare that they have no conflict of interests.
